# Preferential CD8 rather than CD4 T-cell response to wear particles of polyether-ether-ketone and highly cross-linked polyethylene

**DOI:** 10.1039/c7ra10589d

**Published:** 2018-01-09

**Authors:** Zhe Du, Shujun Wang, You Wang

**Affiliations:** Department of Bone and Joint Surgery, Renji Hospital, School of Medicine, Shanghai Jiaotong University 145 Middle Shandong Road Shanghai China 200001 drwangyou@163.com +86 21 53882181 +86 21 53882181; Department of Immunology, Shanghai Institute of Immunology, Shanghai Jiaotong University School of Medicine Shanghai China

## Abstract

The efficacy of polyether-ether-ketone (PEEK) as a bearing material in knee components, a potential alternative to the currently used highly cross-linked polyethylene (HXLPE), has attracted a lot of attention recently. This study aimed to systematically assess the effect of particulate wear debris on CD4 and CD8 T-cell responses. HXLPE and PEEK particles (96% less than 5 μm) were generated by custom cryo-milling and pulverization in liquid nitrogen, and then incubated with blood collected from 25 donors. The phenotypes of the T-cells were systematically analyzed by immunostaining and flow cytometry. For the *in vivo* study, 0.1 mL of each particle suspension (about 1.0 × 10^8^ wear particles) was injected into murine knee joints; the synovium and spleen were collected one week later for histological examination and immunofluorescence staining. PEEK and HXLPE particles did not induce CD4+ T-cell responses; however, CD8+ T-cells might be involved in mediating particle-induced reactions. The T-cell and inflammatory responses induced by PEEK and HXLPE particles were comparable. Further investigations into the frictional properties of materials should be performed to expand on our results.

## Abstract 2

Enriching the understanding of the effects of the particles on the adaptive immune response.

## Graphical abstract

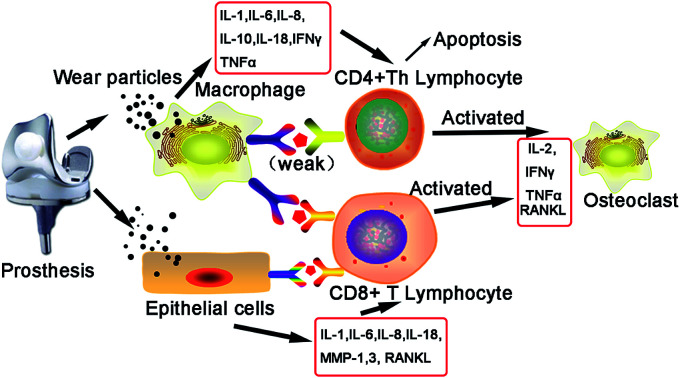

## Introduction

Polyether-ether-ketone (PEEK) and its composites are polymers known to be resistant to fatigue strain. They are radiologically transparent and have mechanical properties suitable for a range of orthopedic applications.^1^ Natural and carbon fiber-reinforced (CFR) PEEK polymers have therefore been used for interbody fusions and dental applications.^2–4^ Highly cross-linked polyethylene (HXLPE) is currently the bearing material of choice for knee arthroplasty.^4,5^ However, the roll-gliding mechanism and differences in the conformity of the articulating surface in the knee can lead to stress in the polyethylene, enhancing the risk of fatigue failure.^4^ Therefore, to improve the durability of knee prostheses, alternative bearing materials with lower wear rates and less biologically active wear particles are required.^6^ Thus, interest in using PEEK as a potential alternative bearing material in the knee has been increasing.

The longevity of total joint arthroplasty is limited by the generation of wear debris and its subsequent biologic sequelae, including aseptic loosening caused by wear particle-mediated inflammation and osteolytic cascades.^7–9^ Particles in the phagocytosable size range (0.1–10 μm) are considered the most biologically reactive, particularly particles with a mean size <1 μm.^1,10,11^ Wear debris have been shown to elicit immune responses with increased inflammatory marker secretion from macrophages.^12^

Beyond the role of macrophages, the mechanism of interaction between the immune system and these wear particles is unclear. Although studies on *in vivo* animal models and *in vitro* cell lines have suggested that PEEK particles generate cytokine expression and histopathological reactions similar to those generated by ultra-high-molecular weight polyethylene (UHMWPE) particles,^1^ the adaptive immune responses of the two particles have not been compared. As PEEK has generated a lot of interest as a potential bearing material in the knee, it is important to assess the immunological reactions of the wear debris produced by PEEK and HXLPE devices.^1^ The aim of this study was to characterize the size and size distribution of particulate wear debris, and to determine its impact on CD4+ and CD8+ T-cell responses. We assumed that the polymer-induced immune responses were mediated by early adaptive immunity and that the two particle types induced comparable adaptive immune responses.

## Materials and methods

### Participants

The study included 25 participants (12 men and 13 women; average age: 62.1 years for men [range, 40–71 years] and 65.7 years for women [range, 44–75 years]). Patients with knee osteoarthritis were recruited, as only these patients may need knee prostheses, and their blood may be exposed to wear debris. Patients with known inflammatory diseases and recent infections were excluded. All procedures were performed in accordance with the guidelines of Shanghai Jiaotong University for use of human samples. All participants provided written informed consent, and ethical approval for the study was received from the Renji Hospital, Shanghai Jiaotong University School of Medicine (grant no. 2017-021).

### Generation of wear particles

Commercially available particles (BioEngineering Solutions, Oak Park, IL, USA) were generated from non-sterilized bulk material from a PEEK tibial tray and an HXLPE insert (Zeniva PEEK ZA-500, Chirulen HXLPE 1020X; Jiangsu Okani Medical Technology Co., Ltd., Soochow, JS, China) using proprietary techniques involving custom cryo-milling and pulverization in liquid nitrogen. The debris was isolated in ethanol and sequentially filtered with polycarbonate membranes. Endotoxins were removed using Pyroclean™. The membranes were washed more than thrice in 70% ethanol, dried in a stock solution of 2 mg/100 μL ethanol, and then tested for endotoxins. Samples with >1 × 10^6^ particles were tested for endotoxins using a limulus amebocyte lysate (LAL) quantitative assay. The particles were sterilized by ethylene oxide (EtO) sterilization with 2000 mg L^−1^ for 1 h at 22 °C, using the AN87 Dosimeter (Warwick, UK).

### Characterization of wear particles

Particle size analysis was performed using scanning electron microscopy (PSEM II Aspex, Pittsburg, PA, USA) and low-angle laser light scattering (LALLS or laser diffraction, Microtrac X-100, Boca Raton, FL, USA) with a liquid sample circulator and an ultrasound dispersal system. The system was flushed 10 times with fresh reverse-osmosis deionized water (>18 Mohm), which was filter-sterilized (0.2 μm filter) to remove potential contaminants. The particle–dH_2_O mixture was added to the liquid circulator at 1 mL s^−1^ to obtain the required laser obscuration value of <3%, as indicated by the automated software. The measurement integration time was 15 s, and 3 repeat measurements were performed for each sample. Wear debris was imaged and analyzed using the aforementioned PSEM II Aspex scanning electron microscope. From the remaining 1–2 mL of enzyme–acid-processed sample, 100–200 μL was vacuum-dried on a polycarbonate membrane (0.1–0.01 μm pores, GTTP, Fisher Scientific). All membranes were dried in a desiccator at 22 °C for 24 h prior to SEM analysis. The number of particles, regardless of size, was counted in at least 15 random image fields on each filter (5 images at each magnification: 1000× and 10 000×). The size and shape of the particles were assessed by technician-operated image analysis of micrographs (Scion Image/NIH Image analysis software). The characterized number of particles in each sample was 450. All particle characterization analyses were performed in accordance with ASTM F1877-05 (Standard Practice for Characterization of Particles).

### Challenging peripheral blood mononuclear cells (PBMCs) with debris

Sixteen milliliters of peripheral blood, with an average of 4 × 10^6^ PBMCs, was isolated from each participant and subjected to density gradient centrifugation. The PBMCs were separated into four groups (two control groups and two experimental groups), and wear debris (20 particles per PBMC) was added to the experimental groups, as described in previous studies.^6,12^ Each type of particle was directly added into the RPMI 1640 medium (Thermo Scientific, Waltham, MA, USA), except for HXLPE, which was premixed with 100% FBS to avoid particle floatation.^13^ The negative control was unchallenged in RPMI 1640 medium. The positive control group was treated with human T-activator CD3/28 (Thermo Scientific). The PBMCs were incubated at 37 °C with 5% CO_2_ for 72 h and then analyzed by flow cytometry.

### T-cell phenotype analysis

T-cell populations were analyzed by immunostaining and flow cytometry, using fluorochrome-conjugated antibodies against CD3 (Pacific Blue™, PB), CD8 (peridinin chlorophyll, PerCP), CD69 (phycoerythrin, PE), interleukin (IL)-10 (phycoerythrin-cyanine 7, PeCy7), IL-17 (PE), IL-2 (allophycocyanin, APC), and interferon (IFN) γ (fluorescein isothiocyanate, FITC). The antibodies were obtained from eBioscience (Thermo Scientific) and BioLegend (San Diego, CA, USA). Fluorescence in the samples was detected and analyzed using a CyAn ADP flow cytometer (Beckton Dickinson) and GraphPad Prism 5 software, respectively.

### Animals

This study was performed in strict accordance with the NIH guidelines for the care and use of laboratory animals (NIH Publication no. 85-23 Rev. 1985) and was approved by the Animal Ethical Committee of the Renji Hospital, Shanghai Jiaotong University, School of Medicine (Shanghai, China). Thirty female Sprague Dawley (SD) rats, each weighing 200–250 g, were obtained. The rats were kept in a surgical research institution for one week before particle injection. They were kept in groups of three per cage and allowed food and water *ad libitum*. They were randomly assigned to one of three treatment groups: control (*n* = 10), PEEK particles (*n* = 10), and HXLPE particles (*n* = 10).

### Surgical procedures

The rats were anesthetized by intraperitoneal injection of ketamine (10 mg kg^−1^). Each rat was immobilized at the knee joint in the maximally flexed position; the right leg was then shaved and depilated. The two types of particle suspensions were sonicated for at least 60 min to avoid particle aggregation before injection. Each particle suspension (0.1 mL; approximately 1.0 × 10^8^ wear particles) was then injected into the right knee of the experimental groups under sterile conditions. PBS (0.1 mL) was injected into the right knee of the control group. After surgery, the rats were housed in ventilated rooms with access to water and food.

### Sample preparation

The rats were sacrificed one week post-surgery. The synovial tissues in the knee joints and spleen tissues were harvested and fixed in 4% buffered formaldehyde for histomorphometric observation and immunofluorescence labeling.

### Histological analysis and immunohistochemistry

After fixation in 4% paraformaldehyde at pH 7.2 for 24 h, the knee synovial membrane was washed with PBS and dehydrated with an automatic dehydrator. After embedding in paraffin, the joint was sliced into 3 μm thick sections and stained with hematoxylin and eosin. Tissue morphology and wear particles were observed under the bright-field microscope and polarizing microscope, respectively. Two sections of each knee synovial membrane were stained immunohistochemically with each primary antibody (anti-CD4, -CD8, -IL-1, -IL-6, and -TNF-α [R&D Systems, Minneapolis, MN, USA]). After staining, the two samples of each primary antibody were evaluated semi-quantitatively with a light microscope at different magnifications (10×, 20×; Carl Zeiss MicroImaging GmbH, Germany). The area labeled in the immunohistochemistry procedure was analyzed using Image-pro plus 6.0 (Media Cybernetics, Inc., Rockville, MD, USA). Six fields of each section at 200× original magnification were digitized and transferred to the Image-pro plus 6.0 software. The area covered by positive cells (brown color) was determined, and the brown-labeled area was then divided by the area occupied by the cells and multiplied by 100.

### Double immunofluorescence staining of CD4+ and CD8+ T-cells in spleen

The primary antibodies were a mixture of two antibodies (anti-CD3 [rabbit antibody] and anti-CD4/CD8 [mouse antibody]). Secondary antibodies were mixtures of Alexa Fluor Cy3-conjugated goat-anti-mouse IgG and Alexa Fluor 488-conjugated goat-anti rabbit IgG antibodies. Cells that showed double staining in the immunofluorescence were manually counted in six fields at 400× original magnification. The number of double-stained cells was then divided by the total number of cells and multiplied by 100.

### Statistical analysis

Data normality was tested before one-way analysis of variance (ANOVA) with Least Significant Difference (LSD) *post hoc t*-tests. Mann–Whitney *U* or Wilcoxon rank sum tests were used for nonparametric data. Differences with *p* < 0.05 were considered statistically significant. The sample size for the *in vitro* study was chosen from a preliminary experiment with 10 donors after a power analysis calculation. For a power of ∼0.9 (*α* = 0.05) a minimum sample size of 20 in each group was required (Fig. 2).

## Results

### Low-angle laser light scattering (LALLS) and scanning electron microscopy (SEM)

**Fig. 1:**
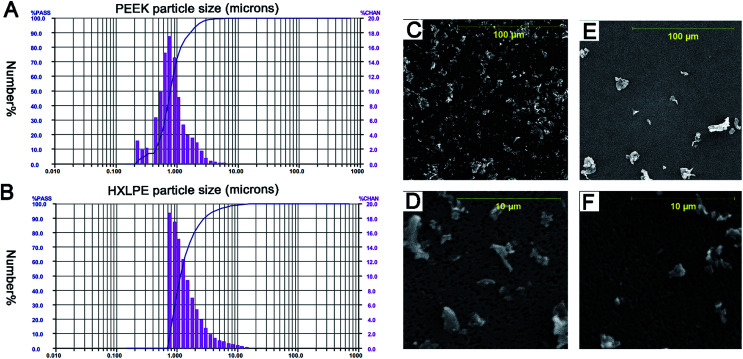
Particle size distributions of PEEK and HXLPE (number-based) obtained using low-angle laser light scattering (LALLS) and scanning electron microscopy (SEM) of the wear particles. Size = equivalent spherical diameter, based on cross-sectional area (μm), % PASS = the cumulative percentage of particles below each size (line), and % CHAN = the relative percentage of particles within each size range (bars). (A) Number-based analysis of PEEK. (B) Number-based analysis of HXLPE. (C and D) SEM images of PEEK wear particles in two magnification fields ((C) magnification 1000×, (D) magnification 10 000×). E and F. SEM images of HXLPE wear particles in two magnification fields ((E) magnification 1000×, (F) magnification 10 000×).

The results of LALLS showed that the mean PEEK particle diameter was 0.94 μm. The size-range of the particles was identified by number analysis software to be 0.24–9.25 μm, with 99% of the particles below 5 μm and 71% at the submicron level (Fig. 1A). The mean HXLPE particle diameter was 1.66 μm; the size-range was 0.82–31.11 μm, with 96% of the particles below 5 μm and 36% at the submicron level (Fig. 1B). The particle sizes showed no significant differences between the two particle types (*p* = 0.06). The results of the SEM showed that the particles were globular or granular at all size ranges (Fig. 1C–F).

**Fig. 2:**
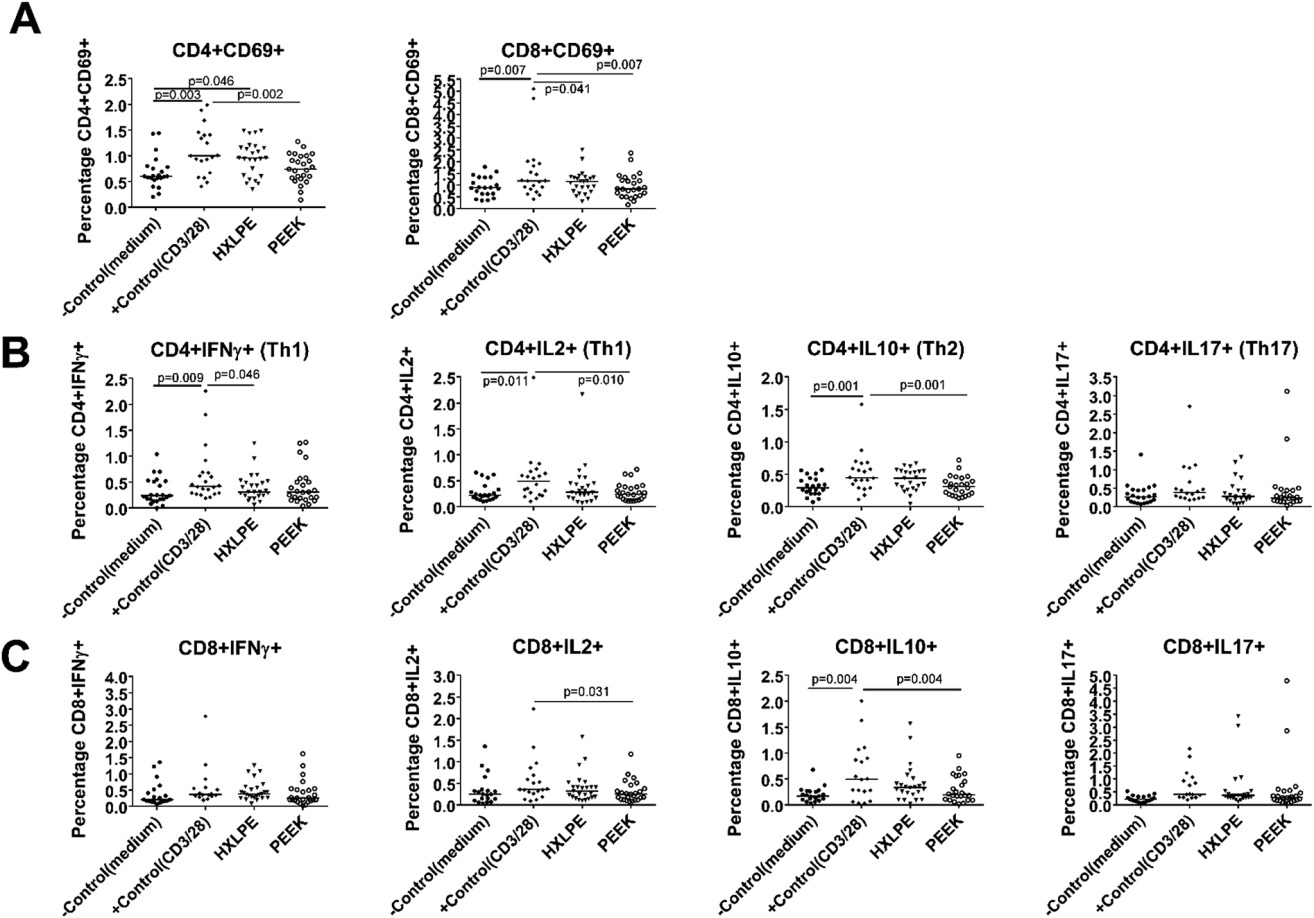
T-cell populations analyzed by immunostaining and flow cytometry in human peripheral blood. Each data point represents the response of an individual subject (*n* = 25), and the bar indicates the median value. As the equivalent values may be overlapped into one point, the cases of some groups appear to be less than 25. (A) T-cell activations identified by immunostaining and flow cytometry. CD4+CD69+ and CD8+CD69+ cells are shown for control cells or cells challenged with HXLPE and PEEK particles. (B) CD4+ T-cell functionality identified by immunostaining and flow cytometry. Th1, Th2, Th17 cells for control cells or cells challenged with HXLPE and PEEK particles are shown. (C) CD8+ T-cell functionality identified by immunostaining and flow cytometry. CD8+IFNγ+/IL-2+/IL-10+/IL-17+ cells for control cells or cells challenged with HXLPE and PEEK particles are shown.

### Effect of wear particles on T-cell activation in human peripheral blood

**Fig. 3:**
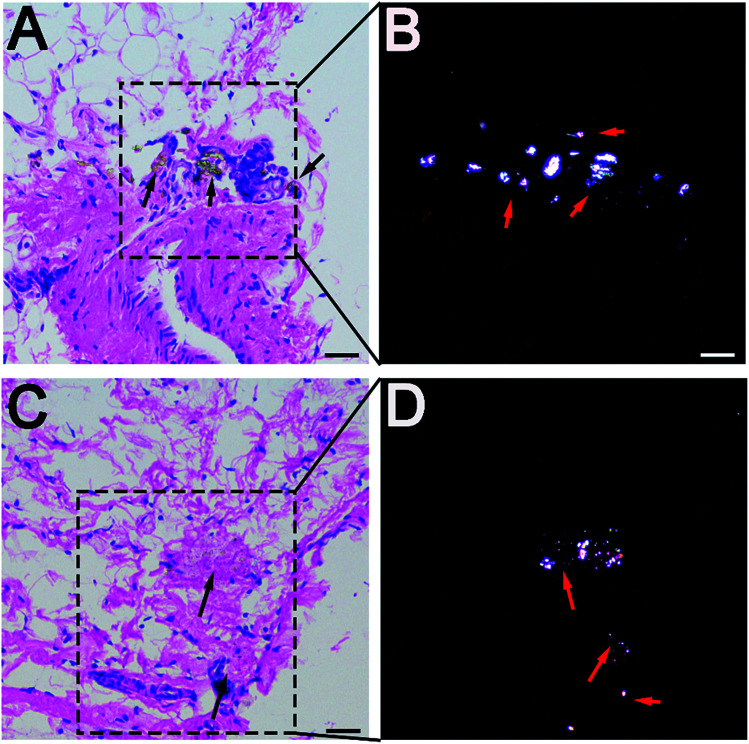
Histological examination of rat synovium tissues. (A and C) Hematoxylin and eosin staining of synovium (A for PEEK, C for HXLPE), indicated by black arrows; wear particles could be found in necrotic tissues. (B and D) Polarizing microscopy of wear particles (B for PEEK, D for HXLPE), indicated by red arrows; wear particles were clearly noted. Bar = 50 μm.

There were no significant differences between the experimental groups (HXLPE and PEEK) and the negative control group in terms of T-cell activation (Fig. 3A). However, HXLPE exhibited a distinct CD4+CD69+ T-cell response, compared to the response of the negative control (*p* = 0.046; Fig. 3A). Compared to that of the positive control, all experimental groups showed lower or comparable responses. For example, PEEK wear debris showed lower CD4/8+CD69+ T-cell responses than did the positive control (*p* = 0.002 and 0.007, respectively). PEEK wear debris was comparable to HXLPE particles in terms of T-cell activation (*p* > 0.05) (Fig. 3A).

### Effect of wear particles on CD4+ T-cell functionality in human peripheral blood

There were no significant differences between the experimental groups (HXLPE and PEEK) and the negative control group in terms of CD4+ T-cell functionality (Th1, Th2, Th17) (Fig. 3B). Compared to those of the positive control, the experimental groups all showed lower or comparable responses. For example, PEEK debris showed lower CD4+IL2+/IL10+ responses than the positive control did (*p* = 0.01 and 0.001, respectively). PEEK wear debris was comparable to HXLPE particles in terms of CD4+ T-cell functionality (Fig. 3B).

### Effect of wear particles on CD8+ T-cell functionality in human peripheral blood

There were no significant differences between the experimental groups (HXLPE and PEEK) and the negative control group in terms of CD8+ T-cell functionality (CD8+IFNγ and CD8+IL2+) (Fig. 3C). Compared to those of the positive control, the experimental groups all showed lower or comparable responses. PEEK debris showed lower CD8+IL2+/IL10+ T-cell responses than the positive control did (*p* = 0.031 and 0.004, respectively). PEEK wear debris was comparable to HXLPE particles in terms of CD8+ T-cell functionality (Fig. 3C).

### Histological and immunohistochemical analysis of rat synovium

**Fig. 4:**
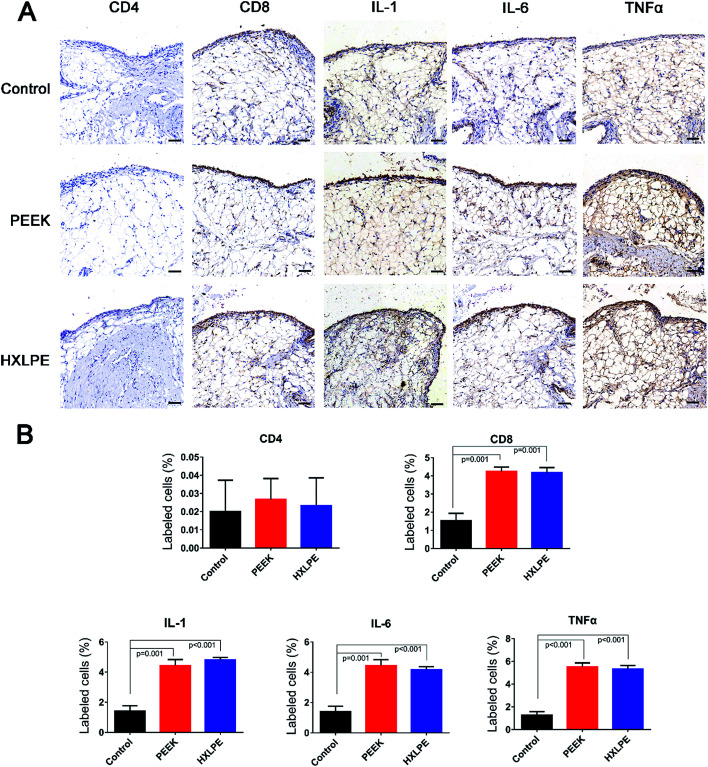
Immunohistochemical staining and analysis of rat synovium. (A) Immunohistochemical staining (CD4, CD8, IL-1, IL-6, and TNFα) of synovium in three groups. (B) Semiquantitative analysis of the positive cells area. Bar = 100 μm.

Wear particles were detected in the synovium (Fig. 3A and C) and could be clearly verified under a polarized light microscope (Fig. 3B and D). The immunohistochemical analysis revealed almost no CD4+ T-cells in the control or experimental groups (Fig. 4). However, CD8+ T-cells were found in the experimental groups, with comparable expression levels in the PEEK and HXLPE groups (Fig. 4). PEEK and HXLPE showed comparable expression of inflammatory cytokines (IL-1, IL-6, and TNFα).

### Immunofluorescence labeling of rat spleen

**Fig. 5:**
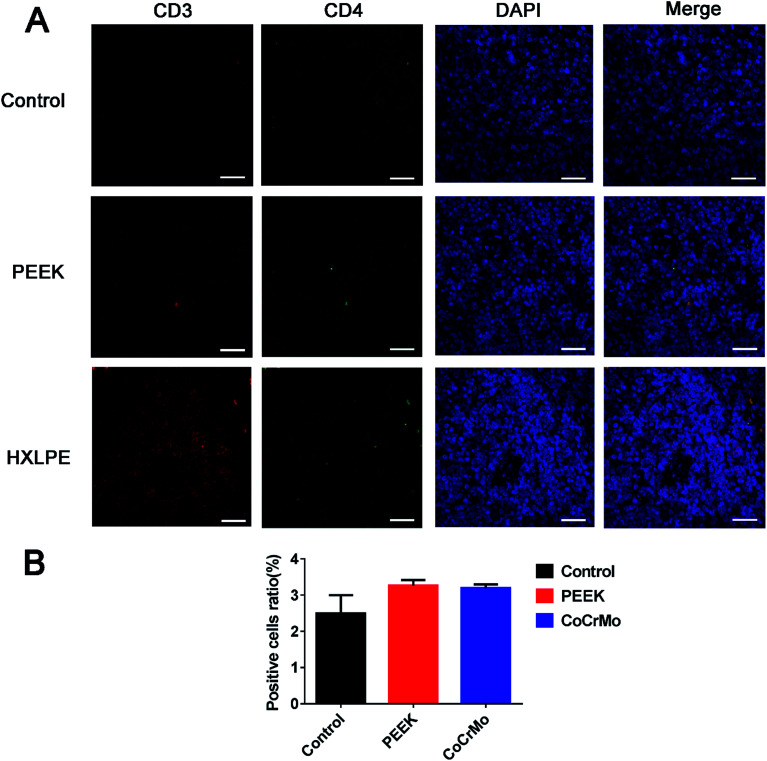
Immunofluorescence labeling of CD4+ T-cell responses in rat spleen. Pictures in white pulps were shown. (A) Immunofluorescence labeling of CD4+ T-cells of spleen in three groups. (B) Analysis of the ratio of positive cells. Bar = 50 μm.

**Fig. 6:**
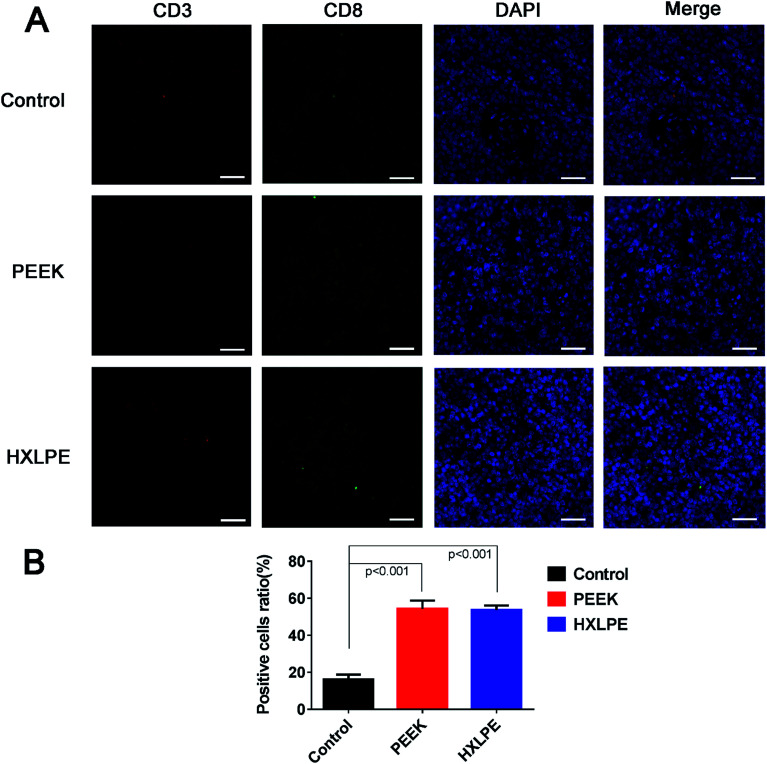
Immunofluorescence labeling of CD8+ T-cell responses in rat spleen. Pictures in white pulps were shown. (A) Immunofluorescence labeling of CD8+ T-cells of spleen in three groups. (B) Analysis of the ratio of positive cells. Bar = 50 μm.

For CD4+ T-cell responses, there were no significant differences between the experimental groups (PEEK and HXLPE) and the negative control group (Fig. 5). For CD8+ T-cell responses, increased numbers of positive cells were found in the PEEK and HXLPE groups, with comparable responses between the two groups (Fig. 6).

## Discussion

For improving the survival rates of knee prostheses, there is a growing demand for alternative bearing materials with lower wear rates and less biologically active wear particles.^1,6,14^ Considering the interest in using PEEK as a potential alternative bearing material in the knee, the biological responses of the debris, especially the immune responses, need to be investigated. In this study, we systematically evaluated the effect of PEEK and HXLPE wear debris on immunological reactions (CD4+ and CD8+ T-cell responses). Our results suggested that PEEK and HXLPE particles did not induce CD4+ T-cell responses, while CD8+ T-cells might be involved in mediating particle-induced reactions. The immunological and inflammatory responses induced by PEEK and HXLPE particles were comparable. Previous reports about PEEK and HXLPE wear particles were confined to studies of inflammatory responses caused by the particles. However, our current study explored the adaptive immune responses, and provided both *in vivo* and *in vitro* experimental data. Enriching the understanding of the effects of the particles on the adaptive immune response was the innovative point of this paper.

Aseptic loosening is the major cause of revision surgery over the mid- and long-term. However, there is a growing consensus that only particles within a phagocytosable range (<10 μm) produce *in vitro* inflammatory responses. In this study, the mean particle size of PEEK and HXLPE was found to be approximately 0.1–10 μm, which is generally considered the most biologically reactive size range.^1,15,16^ In addition, the particle sizes and size distributions of the two particles types were similar, as measured by LALLS and SEM analyses. Lymphocyte activation requires a major histocompatibility complex (MHC)-associated antigen-specific signal, as well as a simultaneous “costimulatory” signal by antigen presenting cells (APCs).^17^ When PBMCs were treated for over 72 h, the debris was initially processed by macrophages, which was followed by downstream activation of T-cells. In local synovial tissues, monocytes were found to participate in the initial macrophage processing of the debris. Furthermore, monocytes and the macrophages are generally responsible for mediating debris-induced inflammation, affecting local cell types (*i.e.*, osteoclasts and osteoblasts) and causing device loosening.^18^ Previous studies have focused more on the innate immune response against wear particles; however, adaptive immune responses mediated by polymer particles have not been well studied.

While debris from metallic implants has been reported to cause a multitude of reactions, such as activation of the adaptive immune system through antigen presentation, release of proinflammatory mediators, cytotoxicity, DNA damage, and oxidative stress, there have been no such reports for debris from polymeric implants.^19^ CD4+ T-cells are specific for antigens presented by MHC II molecules on the surface of APCs, such as macrophages, dendritic cells, and B-lymphocytes. Previous studies have reported that T-helper cells are the T-cell subtype that dominates implant debris-associated responses. Th1 cells are characterized by the secretion of IFNγ and IL-2.^20,21^ The Th2 response is the most effective activator of B-lymphocytes, and it is associated with humoral immunity.^17^ In this study, CD4+ T-cells (Th1 and Th2) were barely induced by either of the polymer wear particles, since neither the PBMC phenotypes nor the positive cells expressed in local tissues/peripheral lymphoid organs in the experimental groups were significantly higher than those in the negative control group. The results indicate that, contrary to the results of previous studies on metal particles, CD4+ T-cells might not be involved in polymer-induced immune responses. The number of CD4+ cells has also been suggested to decrease during strong apoptotic reactions.^22^ Th17 cells, characterized by their secretion of IL-17, play a crucial role in the initiation of inflammation. They recruit neutrophils and macrophages to infection sites, or tissue damage sites in the case of sterile inflammation.^12,23^ Th17 cells are associated with autoimmune diseases; the level of this indicator was lower in the experimental groups than in the negative controls, indicating that the particles are less likely to cause autoimmunity.

CD8+ T-cells are specific for class-I MHC molecules on foreign proteins.^22,24^ A previous study had reported an increased percentage of HLA DR+CD8+ T-cells in patients implanted with metal-on-metal bearings.^25^ However, studies on polymer T-cell HLA reactions are rare. *In vitro*, the expression of CD8-related T-cell phenotypes was lower in the experimental groups than in the negative control group, while *in vivo*, CD8+ T-cell responses were observed in both local synovial tissues and spleen. Summarizing the results of these two studies, the *in vivo* results seem more credible, as the *in vitro* environment was relatively simple, with no complex factors or immune organs involved. The mechanisms by which CD8+ T-cells participate in the response are not clear. It is assumed that the wear particles could induce apoptosis and/or direct necrosis of endothelial cells, and that these necrotic substances and APCs could activate the class-I MHC molecular pathways, resulting in elevated CD8+ T-cell responses.

In bulk form, PEEK composites are generally considered biocompatible.^1^ In previous studies, PEEK-OPTIMA particles were found to be more biocompatible than UHMWPE particles were, as the former induced fewer inflammatory cytokine responses. Thus, compared to UHMWPE, PEEK-OPTIMA implant debris posed little risk of inflammation.^19^ Another study used quantitative immunohistochemistry on epidural tissues and found that unfilled PEEK wear particles did not elicit aggressive immune responses.^2^ In our study, we examined the levels of inflammatory factors in the tissues, and found no differences between the two particle types, which was consistent with other reports in the literature. The inflammatory factors were induced not only by macrophage activation, but also by T-cell activation, especially CD8+ T-cells in our study. Therefore, the expression of inflammatory cytokines was the combined effect of macrophages and T-cells.

The immunological and inflammatory responses induced by PEEK and HXLPE particles were comparable. Therefore, when the size and wear volume of the particles is equal, the chemical properties and wear-resistance of the material itself may be the key factors in evaluating its effect. As different friction pairs show different wear rates, the frictional properties of the two types of materials need to be further explored, with combinations of different friction pairs.

There are many kinds of materials used in artificial joints at present, such as PEEK, HXLPE, titanium alloy, cobalt–chromium–molybdenum alloy, and ceramics. All have been certified for biological safety and use in the human body. However, there is a critical relationship between biological reactions of the material and the size of the particles. In this report, the size of the two materials was similar, so they were comparable. However, it is difficult to control the sizes of other particles during experimentation to allow them to be compared in this manner. However, we will continue to perform more studies to compare a wider array of particles in the future.

There were several limitations to this study. First, the wear condition alone may not fully represent the nature of the wear in clinical cases; thus, further studies using wear particles isolated from tissues or validated joint replacement simulators, instead of manufactured particles, are required. However, the polyethylene wear particles included in this study are similar to those occurring clinically, which have predominantly a spheroid shape and a size inferior to 1 μm (>90%).^26^ Second, nanosized wear particles have been identified both in *in vitro* wear test lubricants and in tissues retrieved during revision surgery;^27^ our study did not include nanosized wear particles, which need to be discussed in future studies. Third, it is still unknown whether the complex environment of the peri-implant milieu ultimately makes cells more susceptible to the effects of *in vivo* particle exposure. The mechanisms by which polymer particles induce CD8+ T-cell responses need to be further investigated.

## Conclusions

In summary, PEEK and HXLPE particles did not induce CD4+ T-cell responses, while CD8+ T-cells might be involved in mediating particle-induced reactions. The T-cell and inflammatory responses induced by PEEK and HXLPE particles were comparable. Further investigations into the frictional properties of materials should be performed in order to expand on our results.

## Conflicts of interest

The authors declare that there are no conflicts of interest.

## Supplementary Material
